# Stomach Temperature Records Reveal Nursing Behaviour and Transition to Solid Food Consumption in an Unweaned Mammal, the Harbour Seal Pup (*Phoca vitulina*)

**DOI:** 10.1371/journal.pone.0090329

**Published:** 2014-02-26

**Authors:** Caroline C. Sauvé, Joanie Van de Walle, Mike O. Hammill, John P. Y. Arnould, Gwénaël Beauplet

**Affiliations:** 1 Département de Biologie, Université Laval, Québec, Québec, Canada; 2 Québec-Océan, Québec, Québec, Canada; 3 Maurice Lamontagne Institute, Fisheries and Oceans Canada, Mont-Joli, Québec, Canada; 4 School of Life and Environmental Sciences, Deakin University, Burwood, Victoria, Australia; Institut Pluridisciplinaire Hubert Curien, France

## Abstract

Knowledge of milk transfer from mother to offspring and early solid food ingestions in mammals allows for a greater understanding of the factors affecting transition to nutritional independence and pre-weaning growth and survival. Yet studies monitoring suckling behaviour have often relied on visual observations, which might not accurately represent milk intake. We assessed the use of stomach temperature telemetry to monitor suckling and foraging behaviour in free-ranging harbour seal (*Phoca vitulina*) pups during lactation. Stomach temperature declines were analysed using principal component and cluster analyses, as well as trials using simulated stomachs resulting in a precise classification of stomach temperature drops into milk, seawater and solid food ingestions. Seawater and solid food ingestions represented on average 15.3±1.6% [0–40.0%] and 0.7±0.2% [0–13.0%], respectively, of individual ingestions. Overall, 63.7% of milk ingestions occurred while the pups were in the water, of which 13.9% were preceded by seawater ingestion. The average time between subsequent ingestions was significantly less for seawater than for milk ingestions. These results suggest that seawater ingestion might represent collateral ingestion during aquatic suckling attempts. Alternatively, as solid food ingestions (*n* = 19) were observed among 7 pups, seawater ingestion could result from missed prey capture attempts. This study shows that some harbour seals start ingesting prey while still being nursed, indicating that weaning occurs more gradually than previously thought in this species. Stomach temperature telemetry represents a promising method to study suckling behaviour in wild mammals and transition to nutritional independence in various endotherm species.

## Introduction

During the period of parental dependence, offspring growth and survival rely on parental nutritional provisioning, mostly determined by maternal milk transfer in mammals [1], [2]. Upon nutritional independence, offspring survival depends on environmental conditions, population density, body reserves and the ability to forage efficiently in a given environment [3], [4], [5], [6], [7]. Such a marked shift of the factors affecting offspring survival makes the transition to nutritional independence a critical process in the life history of wild animals [1], [8], [9].

The study of nursing and foraging behaviour in mammals provides important information related to the transfer of energy from mother to offspring, as well as the transition to nutritional independence. Pinnipeds (phocids, otariids and walruses) are an excellent model for comparative studies of suckling behaviour and transition to nutritional independence since females give birth to single young, phylogeny is acceptably established between families [10], and differential mating and maternal strategies are well documented between the two major families [11]. Among phocids (true seals), females usually fast on land throughout a short (4–50 *d*) lactation period [11], [12]. Weaning is considered to be abrupt in phocids and is followed by a fasting period during which physiological development continues and foraging skills are improved [12], [13], [14], [15]. In otariids (fur seals and sea lions), females alternate between episodes on land primarily for nursing and foraging trips at sea [11], [16]. Otariid pups begin to consume solid food near the end of the lactation period [12], [16], making the transition to nutritional independence more gradual in otariids than in phocids. In spite of these phylogenetic distinctions, females of some phocid species do not follow the typical phocid maternal strategy as they forage in the course of the lactation period [17], [18], [19], [20,]. Accordingly, harbour seal females undertake foraging trips at sea beyond the first week of lactation [19], [21]. This ‘otariid-type’ maternal strategy is thought to be due to the inability of females to store sufficient energy and support milk production throughout the lactation period [11]. Harbour seal pups are also exceptional among phocids because they take to the water soon after birth [22], [23], [24], perform dives usually associated with foraging before weaning [24] and sometimes accompany their mother at sea during foraging trips [23]. However, it is generally considered that weaning in this species is abrupt as in most phocids [25].

Studies investigating suckling behaviour have mostly relied on direct observations [26]. However, visual observations of free-ranging animals are often limited to daylight hours and are affected by weather conditions, position of the observer, and access to nursing sites. Nocturnal and underwater suckling events have been reported in many mammal species (*e.g*., [27], [28], [29], [30]). Moreover, scan frequencies may not be representative of suckling success [31], [32], hindering accurate characterization of nursing patterns. Although the energetics of lactation has been assessed using indirect methods such as isotope dilution and doubly labelled water in several species (*e.g*., [33], [34], [35], [36], [37], [38]), such methods only provide information on average energy transfer between two time points.

**Figure 1:**
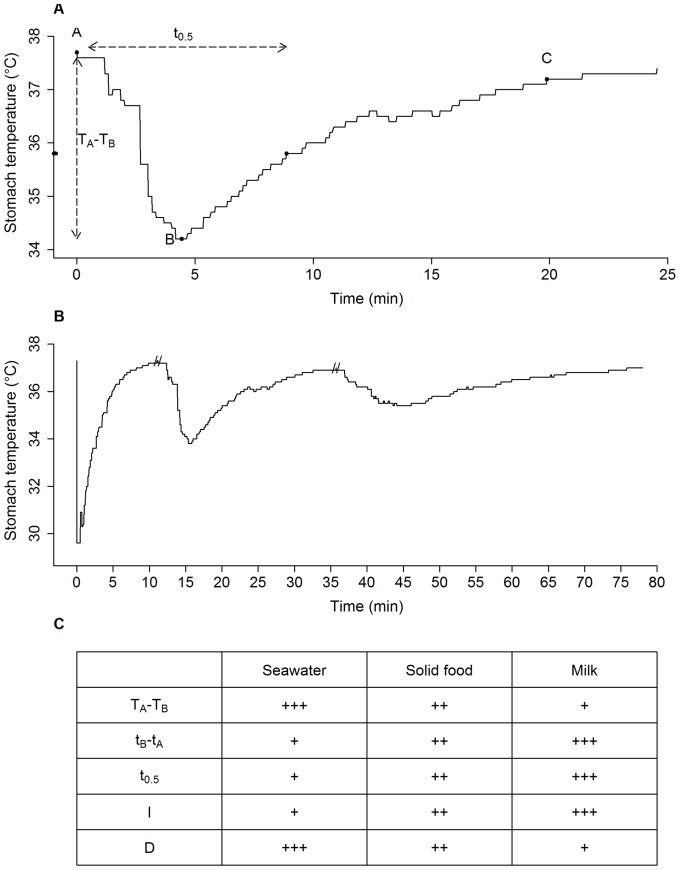
Characteristics of PDERs generated by different ingestion types. A: a PDER event where points *A*, *B* and *C* are showed (see text for definitions), in addition to the amplitude of temperature drop (*T_A_*-*T_B_*), duration from *A* to half-way temperature recovery (*t_0.5_*) and temperature at half-way temperature recovery (*T_0.5_*), indicated by a black point on the Y axis. B: From left to right; PDER events associated with seawater, solid food and milk ingestions, respectively, extracted from a harbour seal pup stomach temperature record. C: Expected order of magnitude for 6 stomach temperature variables.

Stomach temperature telemetry has been developed and broadly used to monitor the foraging behaviour of marine endotherm predators (*e.g*, [39], [40], [41], [42], [43]). Because their body temperature is greater than that of their prey, food consumption by endotherms feeding on cooler items results in a stomach temperature variation characterised by a precipitous drop and subsequent exponential rise (PDER event; [39]). Similarly, mammary glands are situated in the dermis tissue, which is cooler than the animal core temperature (*e.g*., [44]). Suckling therefore results in a PDER event in offspring stomach temperature [28]. Stomach temperature telemetry has proven to be a valuable technique for detecting suckling events in both captive [28] and free-ranging [29] harbour seal pups, representing a promising method to study nursing behaviour in various wild mammals. The interpretation of stomach temperature data from nursing mammals requires a validation technique in order to discriminate the PDER events associated with suckling from those caused by seawater or solid food ingestion. Maternal milk, seawater and ectothermic organisms have different temperatures and/or thermodynamic properties such that their ingestion should yield distinct PDER curve patterns (Fig. 1). Ingestion of maternal milk by seal pups has been shown to result in PDER events of considerably lower amplitude compared to seawater or prey ingestion [28], [29]. Similarly, seawater has greater thermal conductance and lower viscosity than prey items (*e.g.* fish), resulting in PDER events with faster stomach temperature recovery [45], [46]. Accordingly, milk ingestion has been previously distinguished from ingestion of colder materials in harbour seal pups based on the rate of stomach temperature decrease [28], [29]. Likewise, an index of the temperature recovery rate has previously been developed to efficiently discriminate fish consumption from seawater drinking in adult pinnipeds and seabirds [41], [47]. By combining these two methods, discrimination between the three possible ingestion types (milk, seawater and solid food) might therefore be possible in lactating pups. Such discrimination is of primary importance to accurately investigate the nursing behaviour and transition to nutritional independence in mammals.

The main objective of this study was to provide a method to efficiently discriminate the three possible ingestion types (milk, seawater and solid food) in nursing mammals that feed on cooler items. We hypothesized ingestion types could be distinguished based on the characteristics of the PDERs they generate using a combination of multivariate data analysis techniques and laboratory stomach simulations [28], [46]. As seawater ingestion was frequently observed in the stomach temperature records from a small sample of free-ranging harbour seal pups [29], we also tested different hypotheses explaining seawater consumption by pre-weaned pups. We expected that if pups drink seawater when dehydrated, occurrence of seawater ingestion would be positively correlated with the time elapsed since the last milk ingestion. Alternatively, if seawater ingestion represents a consequence of underwater suckling attempts, it would likely be followed by a subsequent ingestion shortly after. Finally, we investigated the prevalence of pre-weaning solid food consumption in harbour seal pups.

## Materials and Methods

### Ethics statement

Animal handling procedures were approved by the Animal Care Committees of Université Laval (permits numbers: 200844 and 2011020) and of Fisheries and Oceans Canada (permits numbers: IML 2010-030, IML 2011-004, and IML 2012-003).

### Animal handling and instrumentation

This study was conducted on the Bic Island harbour seal colony (48°24′ N; 68°51′ W), located around a private island in the St. Lawrence River estuary, during the 2010–12 breeding seasons. Harbour seal pups were captured with a long dip net [48] throughout the lactation period (from mid-May to July). At first capture, pup sex, umbilicus state and mass (±0.5 kg; Salter spring scale, West Bromwich, England) were recorded. To facilitate recaptures, individuals were marked with a coloured and numbered head tag (Seal Hat, Dalton, England) glued (Loctite #422 cyanoacrylate glue and #7452 Accelerator, Loctite Corp., Mississauga, ON, Canada) on the head and tagged with a unique numbered tag in a hind flipper (Jumbotag, Dalton, England). Recaptures of pups were performed as often as possible until weaning, with a 48 h minimum interval between successive captures.

As most pups were not captured immediately after parturition, two indirect methods were used to estimate pup age (d). In the case of very young pups, age was determined according to the umbilicus degeneration stages [48], [49], [50]. In older pups, individuals were weighed more than once during the season and age was determined by back-calculation to birth mass using a mean growth rate for the cohort (detailed in [51]).

To monitor stomach temperature changes, free-ranging pups were equipped with stomach-temperature pills (STP; non-modified STP3: 32 g, 6.3 cm ×2.2 cm; Wildlife Computers Inc., Redmond, WA, USA) introduced by intubation using a lubricated (K-Y Jelly, Johnson & Johnson, New Brunswick, NJ, USA) 2.54 cm diameter flexible tube inserted into their stomach [29]. Every 10 s, the STP data were transmitted to a time-depth recorder (TDR; MK10-L, Wildlife Computers Inc.) glued (5-Cure Marine Epoxy, Industrial Formulators, Auburn, WA, USA) to the pup's dorsal fur [29]. We performed laboratory calibration tests of STPs (see [28] for protocol details) that indicated an average response time of 18±2 s (range: 10–27 s) due to the delay between STP emission and TDR reception of temperature data. In addition, to facilitate relocation for recapture, a VHF transmitter (3PN, Sirtrack ltd., Havelock North, New Zealand) was also glued on the pup's neck. Data from devices still attached to the animal were downloaded in the field upon each recapture using a portable computer. Handling time rarely exceeded 15 min from capture to release.

### Data processing, analysis and stomach temperature simulations

All data processing and statistical analyses were performed within the R environment [52], unless stated otherwise. Stomach temperature data (±0.1°C) were extracted from TDRs using the Mk10host v1.25.2006 software (Wildlife Computers Inc.). Since important intra- and inter-individual variations in stomach temperature records prevented the use of automated methods to identify ingestions, the PDER events were identified visually. A scan of stomach temperature plotted against time (using Instrument Helper v3.0, Wildlife Computers Inc.) was performed on each file to locate PDER events, defined as sharp drops in stomach temperature of at least 2 standard deviations from mean temperature calculated over the preceding 10 min, followed by an exponential-shaped temperature increase. PDER event characteristics have previously been described for seawater, prey (in adults) and milk ingestions (*e.g*., [28], [29], [40], [47], [53]). Each PDER event was therefore preliminarily assigned to an ingestion type (*i.e.* milk, seawater or solid food) based on the amplitude of decrease and shape of the recovery curve (convex, concave or pseudo-linear; associated with milk, solid food and seawater ingestions, respectively) viewed in the temperature records (Fig. 1B). Although subjective, this categorising served as a reliable criterion of comparison for the results from following objective classification methods.

To statistically classify the temperature drops into the different ingestion types, three points were defined for each PDER event (Fig. 1A; [47], [53]): (*A*) the point preceding the onset of temperature decrease (*i.e*. before temperature decreases to or below the previous mean temperature (calculated over 10 min) minus 2 standard deviations (s.d.); [42]); (*B*) the point where the lowest temperature is reached; and (*C*) the point after which temperature reaches stability (±1 s.d.) for at least 10 minutes. Six variables were subsequently computed for each PDER event: (1) amplitude of temperature drop, *T_A_-T_B_*; (2) duration of temperature drop, *t_B_-t_A_*; (3) temperature at half-way temperature recovery, *T_0.5_* where
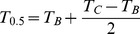
(1)(4) duration from point *A* to half-way temperature recovery, *t_0.5_*; (5) an index of temperature recovery rate, *I* [41], [47] where
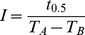
(2)and (6) rate of temperature decrease, D where


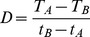
(3)

The PDER events that were difficult to distinguish from natural body temperature variations were noted as low confidence PDER events. These include temperature drops of amplitudes smaller than 0.7°C, or temperature drops presenting similar decrease and increase rates or other characteristics not fully complying with the PDER event definition [39], [46]. Because of their unusual shape characteristics, the previously detailed variables derived from low confidence PDER events might not be representative of typical PDER events. Similarly, when more than one stomach temperature drop occurred before stability was reached, the overlapping PDER events were treated together and defined as multiple PDER events [46]. For these particular events, points *A*, *B* and *C* only characterized the larger drop of the bout. However, Wilson *et al*. [46] found that the STP response was less variable during the first ingestion compared to the subsequent ingestions of the bout. Therefore, the variables computed for multiple PDER events are not representative of typical PDER events either and should be considered separately. Clustering methods are highly affected by outliers. To ensure the formation of PDER event groups that accurately reflect the natural variations in ingestion behavior, low confidence and multiple PDER events were excluded from the cluster analysis.

Using only the retained PDER events, a principal component analysis (PCA) was performed on the centered and scaled values of the 6 variables to generate uncorrelated factors on which a varimax rotation (package *psych*; [54]) was applied. Factors with eigenvalues >1 (Kaiser's criterion) were retained and their scores were used to execute a hierarchical, complete-linkage cluster analysis. This approach allowed the determination of the appropriate number of clusters present in the dataset using the fusion level values criterion [55] as well as the identification of the cluster centers. These centers were introduced as initial seeds in a non-hierarchical K-means cluster analysis that was iterated until the center values became constant [56]. The categorisation of the PDER events by the K-means clustering procedure were compared to their subjective visual classification to determine whether the different groups obtained coincided with a specific ingestion type (*i.e.* milk, seawater, or solid food).

To further increase our efficiency to discriminate between the consumption of water and solid food, ingestions were artificially simulated. A STP was inserted into a balloon and immersed in a circulating water bath kept at a mean temperature simulating the pup's stomach (37.8°C; [28]). Water or fish (cut, non-eviscerated herring) were kept at 12.0°C, the highest water temperature recorded in the study area during the lactation period (St. Lawrence Global Observatory, Rimouski Station, 2010–2012). Items were then injected into the balloon using a 530 mL suction gun and remained in place while they warmed to the temperature of the water bath. To mimic the temperature drop patterns expected in a free-ranging pup's stomach, simulations were made using different quantities of water (25–125 g) and fish (30–300 g). Since the aim of these simulations was to highlight differences in the characteristics of temperature drops generated by water and fish ingestions, the absolute rate of material warming was not important. No attempt was made to imitate the heat conductivity and mixing patterns of a pup's stomach. Consequently, PDER events from laboratory simulations were characterized by greater temperature drops followed by slower warming than those that would be recorded in living animals. Since differences in PDER events were detected in simulating seawater and fish ingestions, relative comparisons between these different ingestion types could nonetheless be applied to PDER events extracted from free-ranging pups' recordings.

**Table 7:** Average time between consecutive ingestion events recorded in nursed harbour seal pups.

	Milk ingestions	Seawater ingestions	*Rand-test* for difference in means
Mean time elapsed since last ingestion (h)	7.5±0.2	6.3±0.5	*P* = 0.007
Mean time until next ingestion (h)	7.8±0.3	4.5±05	*P*<0.001
Mean time until next milk ingestion (h)	8.7±0.3	6.5±0.5	*P*<0.001

For every experimentally-generated PDER event, the 6 variables described above were calculated, centered and scaled, and introduced into a linear discriminant analysis (LDA; package *MASS*; [57]) in which the quantity (g) of injected material was added as a 7^th^ variable. The variable with the highest linear discriminant coefficient was considered as the best predictor of the material (water or fish) that caused the temperature drop [58]. A distribution plot of that particular variable was then produced for the PDER events recorded on free-ranging pups (except low confidence and multiple PDER events). This allowed the identification of discontinuities in the distribution that reflected different ingestion types. PDER events were thereafter classified based on their relative position within the distribution of the best predictor variable of the two non-milk ingestion types (seawater or solid food).

The resulting classifications from both methods, combined with the preliminary visual classification, were compared and pooled to form the three final groups named according to the calculated ingestion type they represented (*i.e*. milk, seawater and solid food). These final categories were used as grouping factors to perform a LDA on the classified (*i.e* single and high confidence) PDER events using the same 6 explanatory variables previously used in cluster analysis. The predictive function generated by the LDA was subsequently used to classify the low confidence and multiple PDER events previously excluded from both classification methods. This procedure led all PDER events to be classified as milk, seawater, or solid food ingestions.

Several factors were investigated to explain seawater and solid food ingestion in pre-weaned pups. Individual growth rate was calculated as the coefficient of a general linear model on mass and age before the estimated weaning age (age ≤33 d; [48]). The ‘hauled-out’ or ‘in water’ status of the pups was determined for each characterized PDER event using the wet/dry threshold of their TDR. The time elapsed since the last milk ingestion, time elapsed since the last ingestion regardless of ingestion type, time elapsed until next milk ingestion and time elapsed until next ingestion were computed for each PDER event and the means compared for each ingestion type.

When appropriate, normality was tested with the Shapiro-Wilk test and homogeneity of variance with a Levene test on means (package *car*, [59]). When the normality criterion was not respected, randomization tests (rand-test; modified from [60]) with 5000 permutations were used rather than *t-tests* to compare statistics between groups. Results were considered significant at P<0.05 and are presented as means ± standard errors (s.e.), unless stated otherwise.

## Results

### PDER events in free-ranging pups and their preliminary classification

A total of 40 free-ranging pups were instrumented. Of these, 35 provided usable data (Table 1) resulting in a total duration of 565 d of stomach temperature recording, with an individual mean duration of 16.1±0.9 d and a total of 1776 PDER events. The overall mean number of PDER events per day per pup was 3.1±0. 3, with substantial inter-individual variations (range: 0.6–4.7 PDER events per day). Among the identified PDER events, 354 exhibited amplitudes <0.7°C (but ≥0.3°C) while 142 had unusual curve shapes and, thus, were classified as low confidence PDER events. Additionally, 13 were considered as multiple PDER events, resulting in 1267 single and high confidence PDER events.

**Table 1:** Characteristics of harbour seal pups (*n* = 40) equipped with STPs during the 2010–2012 nursing periods at Bic Island, Quebec, Canada.

Pup ID	Sex	Cohort	Growth rate (kg/d)	Age at equipment (d)	STP record duration (d)	No. of PDER events
B01	M	2010	0.68	3	TDR not recovered	NA
J09	M	2010	0.53	9	16	57
J10	M	2010	0.48	9	16	69
J11	M	2010	0.53	8	16	58
J12	M	2010	0.59	18	22	55
J29	M	2010	0.40	14	14	59
J32	F	2010	0.63	4	12	41
J33	F	2010	0.42	9	18	40
J35	F	2010	0.49	13	18	40
J42	M	2010	0.67	4	18	17
J44	F	2010	0.45	3	18	36
V01	M	2010	0.75	11	14	47
O04	M	2011	−0.25	4	Equipment failure^1^	NA
O08	M	2011	0.51	18	10	24
O14	M	2011	0.36	11	Equipment failure^1^	NA
O25	M	2011	0.36	14	14	33
O30	M	2011	0.56	4	18	46
O32	F	2011	0.41	8	22	52
O39	F	2011	0.42	2	20	71
O42	M	2011	0.37	12	18	79
O43	M	2011	0.46	8	22	56
O46	F	2011	0.51	8	8	13
O49	F	2011	0.48	9	28	133
O52	F	2011	0.42	15	23	80
O61	M	2011	0.41	17	16	68
O63	F	2011	0.40	8	18	67
O64	M	2011	0.54	7	22	66
O68	M	2011	0.57	12	24	98
O69	F	2011	0.39	8	9	32
O72	F	2011	0.39	6	22	58
O77	F	2011	0.49	10	15	66
J45	M	2012	0.13	Unknown	15	53
J51	F	2012	0.30	8	Mortality^2^	NA
J60	M	2012	0.61	7	15	45
J62	F	2012	0.48	16	10	31
J69	M	2012	0.57	13	11	15
J71	M	2012	0.50	12	TDR not recovered	NA
J72	F	2012	0.62	8	10	30
J74	M	2012	0.71	10	3	14
J99	F	2012	0.68	9	9	27

1 Stomach temperature records empty, either due to pill transmission failure or pill lost by animal.

2 Found dead on the shore following an episode of extreme wave conditions.

Characteristics of the component factors obtained from the PCA are presented in Table 2. According to Kaiser's criterion, 2 factors from the PCA explained 80.3% of total variance and were subsequently retained. These factors were introduced into the varimax rotation, which did not affect the proportion of total variance explained, but equalized its partition between both factors. While one factor exhibited important loadings for the variables related to the duration of the PDER events, the variables associated with temperature decrease heavily loaded on the other factor (Table 3). This indicates that the two factors obtained scored for different variables in accordance with the PDER events' axis characteristics. Furthermore, maximum-likelihood factor analysis revealed that the two factors stemming from the rotated matrix were sufficient in the model (fit  = 0.98, *χ^2^* = 662.55, *P*<0.001), supporting their retention for further analysis. The absolute values of the standardized loadings were >0.30 on at least one factor for the six variables, indicating that all of them were relevant in the PCA (Table 3; [61]).

**Table 2:** Summary of PCA on 6 stomach temperature variables associated with PDER events (*n* = 1269) recorded in nursed harbour seal pups.

Component factor	1	2	3	4	5	6
**Eigenvalue**	1.92	1.06	0.81	0.59	0.34	0.23
**Proportion of variance explained**	0.62	0.19	0.11	0.06	0.02	0.01
**Cumulative proportion of variance explained**	0.62	0.80	0.91	0.97	0.99	1.00

**Table 3:** Factor matrix from varimax-rotated component analysis on PDER events (*n* = 1267) recorded in nursed harbour seal pups.

	Standardized factor loading		
Variable	Factor 1	Factor 2	Communality
*T_A_-T_B_* (°C)	−0.25	−0.93	0.93
*t_B_-t_A_* (s)	0.88	0.17	0.81
*T_0.5_* (°C)	0.21	0.93	0.91
*t_0.5_* (s)	0.93	0.26	0.92
*I* (s°C^−1^)	0.77	0.41	0.77
*D* (°Ch^−1^)	−0.23	−0.66	0.48
Eigenvalue	2.39	2.42	
Proportion of variance explained	0.40	0.40	
Cumulative proportion of variance explained	0.40	0.80	

**Figure 2:**
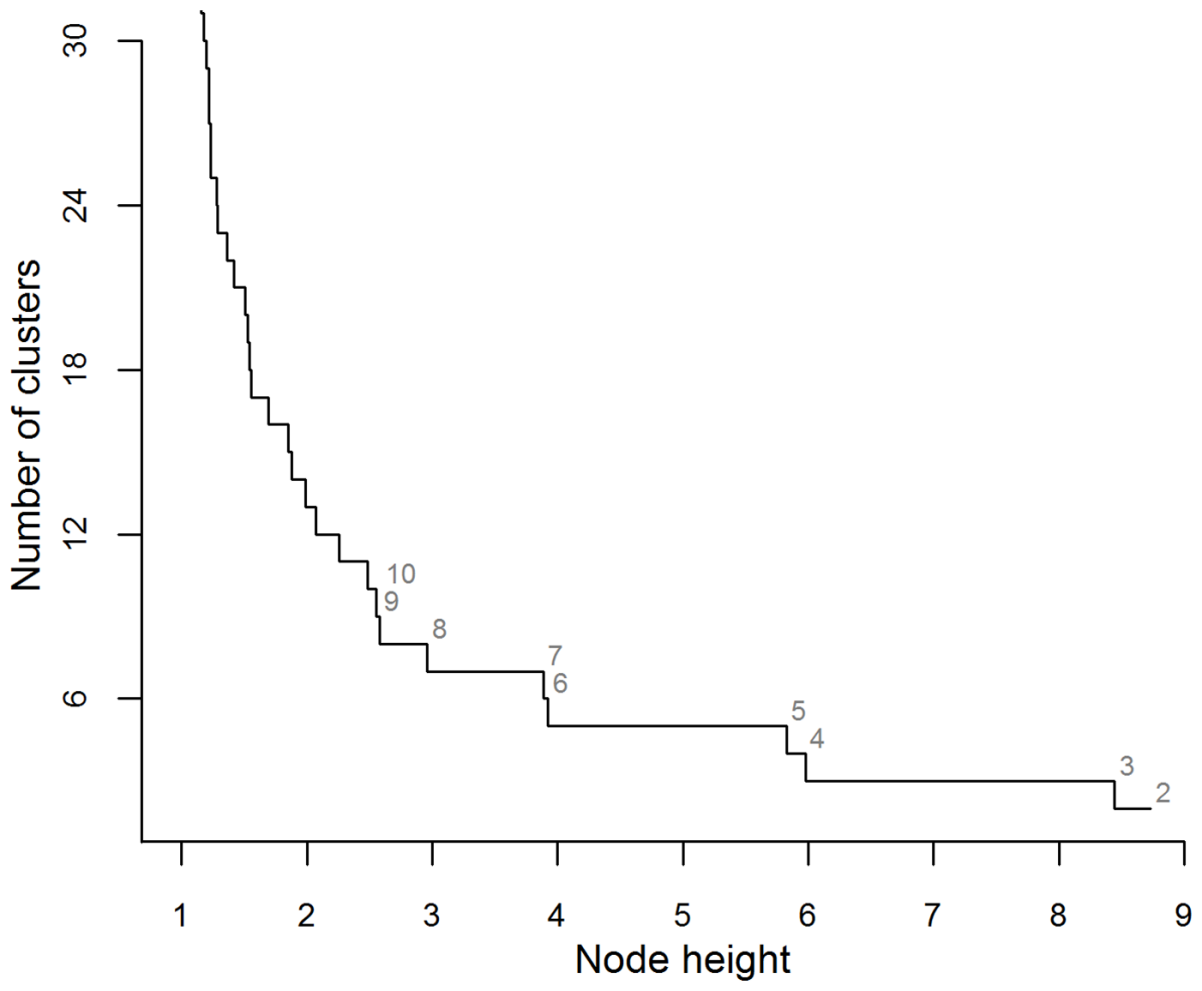
Fusion level values from the complete-linkage hierarchical cluster analysis on PDER events (*n* = 1268). Five groups have been retained for further non-hierarchical cluster analysis since *k* = 5 is the first value of *k* where a small jump in node height was observed.

**Figure 3:**
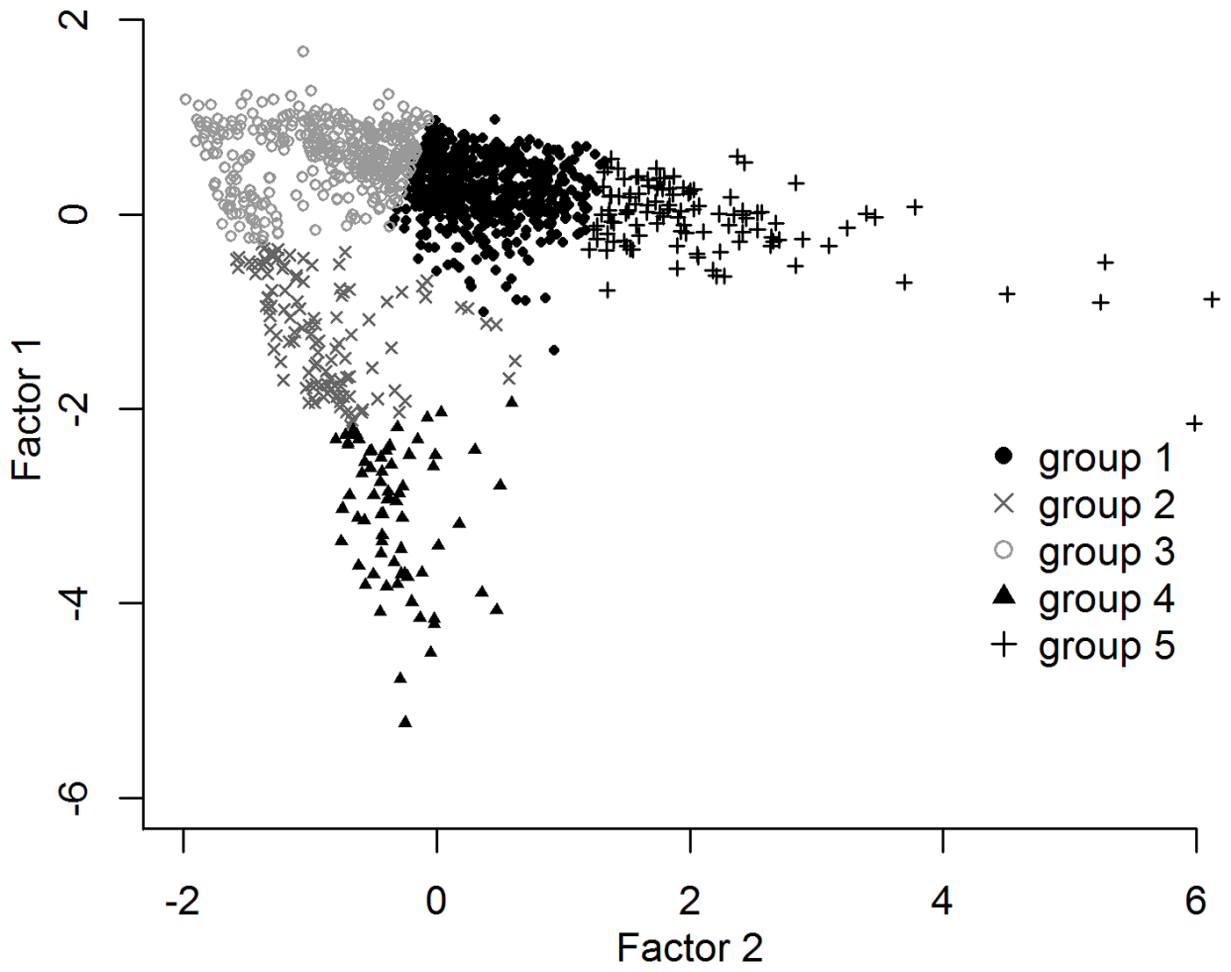
Classification of the PDER events (*n* = 1268) by the K-means cluster analysis. The number of groups was set to *k* = 5 and the temperature variables' means from each group from the complete-linkage hierarchical clustering procedure were used as initial seeds. When compared to the preliminary visual classification, groups 1, 3 and 5 mostly contained PDER events deemed milk ingestions, while groups 2 and 4 were mainly composed of PDER events deemed seawater or solid food ingestions.

Scores for the two factors for each of the 1267 PDER events were calculated from the rotated matrix and introduced as variables in the hierarchical complete-linkage cluster analysis. According to the fusion level value criterion, the optimal number of clusters present in the data was *k* = 5, because it was the first value of *k* before which a small jump in the plot of node height against number of clusters was observed (Fig. 2; [55]). The hierarchical cluster analysis dendrogram was thus cut at *k* = 5, and the means of component factors for each cluster were used as initial seeds in a non-hierarchical K-means cluster analysis (Fig. 3).

When the PDER events from the resulting groups were compared to their preliminary visual classification, three groups contained a high percentage of PDER events identified as milk intakes (group 1: 87.7%, group 3: 99.6% and group 5: 100%; Fig. 3), while two groups were predominantly composed of events identified as either water or solid food intakes (group 2: 99.2% and group 4: 100%). The cluster analysis did not allow for discrimination between seawater and solid food ingestions, but both ingestion types could efficiently be differentiated from milk intakes, with an overall discrepancy of 3.9% between the multivariate analysis and the visual classification method.

### Ingestion simulations and corrected classification of PDER events

**Figure 4:**
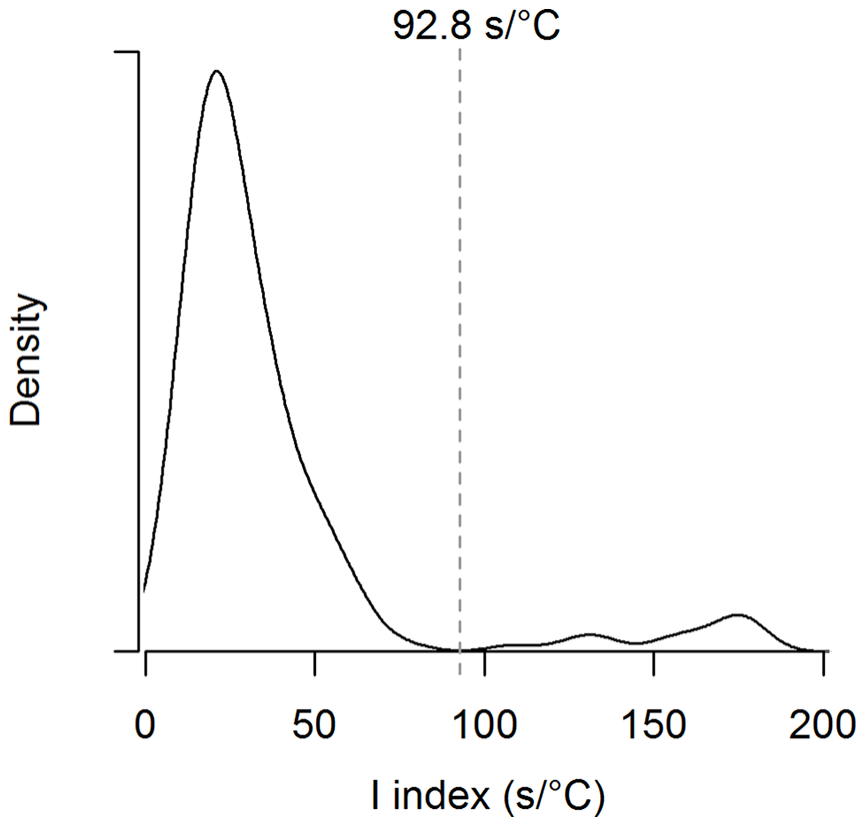
Distribution of the *I* index of PDER events (*n* = 190) from groups 2 and 4. When compared to preliminary visual classification, 98.9% of the PDER events having *I* index values lower than 92.8 s°C^−1^ had been deemed seawater ingestions, while 92.3% of the PDER events with *I* index values greater than 92.8 s°C^−1^ had been deemed solid food ingestions.

All injections of water and fish in the simulated stomachs resulted in sudden drops followed by an exponential rise of temperature recorded by the STP. Resulting coefficients of linear discriminants from the LDA on 7 variables (Table 4) identified the *I* index as the most efficient group predictor. The *I* index computed for water injections was significantly (rand-test, *P* = 0.002) lower than that for fish injections. Therefore, the *I* index's distribution was plotted for the PDER events classified in groups 2 and 4 (*n* = 195; water and solid food ingestions). As shown in Fig. 4, an obvious discontinuity was present in the distribution, with a density equal to zero for *I* values ranging from 77.3 to 108.3 s°C^−1^. The preliminary visual classification of the PDER events from groups 2 and 4 having *I* values >92.8 s°C^−1^ (middle point of the null range of the *I* distribution, see Fig. 4; *n* = 13) indicated that they likely arose from solid food ingestions, while PDER events having *I* values <92.8 s°C^−1^ were mostly visually classified as seawater ingestions.

**Table 4:** Summary of the linear discriminant analysis on 7 stomach temperature variables from water and fish ingestions (*n* = 11) in a simulated harbour seal pups stomach.

		Group mean ± s.e.	
Variable	Coefficient of linear discriminants	Water	Fish
*T_A_-T_B_* (°C)	6.13	11.9±0.3	15.8±0.5
*t_B_-t_A_* (s)	−6.83	70±20	250±60
*T_0.5_* (°C)	1.45	34.0±0.4	33.0±0.6
*t_0.5_* (s)	−9.14	270±20	800±200
*I* (s°C^−1^)	17.57	22.7±0.7	51±7
*D* (°Ch^−1^)	−1.08	705.0±0.5	259.9±0.2
Quantity	−4.13	70.8±0.3	125.0±0.7

Since milk ingestions are expected to produce greater *I* values compared to solid food ingestions (see Fig. 1B), PDER events from all groups having *I* values lower than 92.8 s°C^−1^ (*n* = 251) were classified as seawater ingestions. In contrast, PDER events with *I* values greater than 92.8 s°C^−1^ were categorized as solid food ingestions (*n* = 13) when assigned to groups 2 or 4, and as milk ingestions (*n* = 1003) when assigned to groups 1, 3 or 5 by the cluster analysis.

The resulting linear discriminants from the LDA performed on the 1267 PDER events classified as high confidence and single are presented in Table 5. The resulting predictive function allowed the classification of the remaining 517 multiple or low confidence PDER events with 98.3% of accordance with preliminary visual classification. As a result, all 1776 PDER events were classified as either milk (*n* = 1478), seawater (*n* = 279) or solid food ingestions (*n* = 19). The overall discrepancy in group attribution between the combined statistical analysis and the preliminary visual classification was 2.4%, and was mostly due to PDER events from group 1 (Fig. 3) that were visually considered as solid food ingestions, although classified as milk ingestions by the statistical method.

### Ingestion categories and consumption behaviour

While seawater and solid food ingestions represented 15.7% and 1.1% of all the PDER events, respectively, the relative importance of each ingestion type varied considerably among individuals (Table 6). Not surprisingly, milk represented the most frequent ingestion type for all pups, with the relative proportion of total individual ingestions ranging from 60.0–100% (mean  = 84.1±1.7%). Seawater ingestion across individuals ranged from 0–40.0% (mean  = 15.3±1.6%) of PDER events, while solid food represented 0–13.0% (mean  = 0.7±0.2%) of total ingestions among individuals. All but one individual ingested seawater at least once during the recording period. A total of 19 solid food ingestions were detected among 7 pups. Among these pups, 9 solid food ingestions were recorded in a single individual while among the other 6 animals solid food was ingested on ≤3 occasions.

**Table:** **Table 5.** Summary of the linear discriminant analysis on 6 stomach temperature variables for PDER events (n = 1268) resulting from ingestion in nursed harbour seal pups.

	Coefficient of linear discriminants		Group mean ± s.e.		
Variable	LD1	LD2	Milk	Water	Solid food
*T_A_-T_B_* (°C)	1.794	−0.710	1.18±0.03	5.32±0.2	4.2±0.3
*t_B_-t_A_* (s)	−0.015	−0.520	340±7	59±9	270±30
*T_0.5_* (°C)	0.425	−1.161	37.18±0.02	35.34±0.09	35.4±0.2
*t_0.5_* (s)	−1.063	1.611	702.8±10	160±10	700±30
*I* (s°C^−1^)	0.377	−1.134	810±20	35±2	200±20
*D* (°Ch^−1^)	0.204	−0.295	20±3	1000±90	67±6

Overall, pups were in the water during 63.7% of milk ingestions, of which 13.9% were preceded by a seawater ingestion event. Almost all (98.5%) of seawater ingestions occurred while pups were in the water. Moreover, pups were usually still in the water when performing the subsequent ingestion following a seawater ingestion (98.6% and 72.2% when these ingestions were seawater or milk, respectively). Interestingly, pups that ingested solid food exhibited a greater occurrence of aquatic nursing (milk ingestions while the pup was in the water) than pups that did not ingest solid food (P<0.001; means  = 80±4% and 54±6%, respectively).

**Figure 5:**
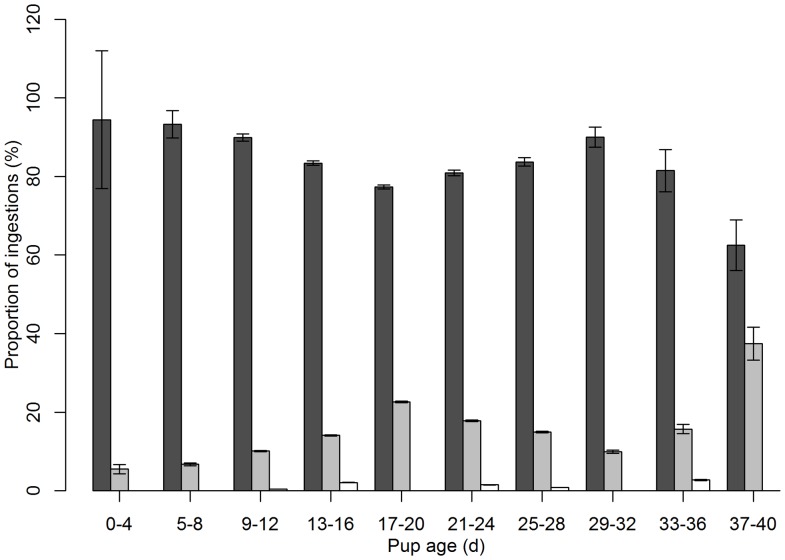
Relative occurrence of each ingestion type as a function of pup age. PDER events (*n* = 1723) from pups of known age (*n* = 34) were used to calculate the proportion of ingestions. Dark-grey, light-grey and white bars represent milk, seawater and solid food ingestions, respectively. Data are presented as means ± s.e.

Milk ingestion occurred throughout the period of stomach temperature monitoring, whereas the first identified consumption of seawater and solid food occurred at 4 and 12 days of age, respectively. As illustrated in Fig. 5, the proportion of ingestions associated with suckling decreased from 94.4% at day 1 to 77.4% at day 20, and further increased up to 90.0% until the estimated weaning age (day 33; [48]). The decreasing proportion of milk ingestions observed in the three first weeks post-partum coincided with a gradual 3-fold increase in the proportion of seawater ingestion events within this period. Solid food ingestions were sparsely distributed throughout the 2 last thirds of the lactation period (Fig. 5).

The daily frequency of seawater ingestion was not correlated to individual growth rate during lactation (*R^2^* = 0.0038, *P* = 0.72). Average times between consecutive ingestions are presented in Table 7.

**Table 6:** Distribution of the PDER events (*n = *1776) recorded in nursed harbour seal pups (*n* = 35) equipped with TDRs and STPs among individuals and ingestion types.

Headtag ID	Total no. of ingestions	Total daily ingestion frequency (No./d)	No. milk ingestions	Daily milk ingestion frequency (No./d)	No. seawater ingestions	Daily seawater ingestion frequency (No./d)	No. solid food ingestions	Daily solid food ingestion frequency (No./d)
J09	57	3.6	50 (87.7)^a^	3.1	7 (12.3)^a^	0.4	0 (0.0)^a^	0.0
J10	69	4.3	49 (71.0)	3.1	11 (15.9)	0.7	9 (13.0)	0.6
J11	58	3.6	55 (94.8)	3.4	3 (5.2)	0.2	0 (0.0)	0.0
J12	55	2.8	48 (87.3)	2.4	7 (12.7)	0.4	0 (0.0)	0.0
J29	59	4.2	47 (79.7)	3.3	11 (18.6)	0.8	1 (1.7)	0.1
J32	41	3.4	40 (97.6)	3.3	1 (2.4)	0.1	0 (0.0)	0.0
J33	40	2.2	35 (87.5)	2.0	5 (12.5)	0.3	0 (0.0)	0.0
J35	40	2.2	37 (92.5)	2.1	3 (7.5)	0.2	0 (0.0)	0.0
J42	17	1.0	16 (94.1)	0.9	1 (5.9)	0.1	0 (0.0)	0.0
J44	36	2.0	34 (94.4)	1.9	2 (5.6)	0.1	0 (0.0)	0.0
V01	47	3.4	39 (83.0)	2.8	8 (17.0)	0.6	0 (0.0)	0.0
O08	24	2.4	16 (66.7)	1.6	8 (33.3)	0.8	0 (0.0)	0.0
O25	33	2.3	29 (87.9)	2.0	4 (12.1)	0.3	0 (0.0)	0.0
O32	52	2.4	47 (90.4)	2.1	5 (9.6)	0.2	0 (0.0)	0.0
O30	46	2.6	39 (84.8)	2.2	7 (15.2)	0.4	0 (0.0)	0.0
O39	71	3.5	62 (87.3)	3.1	9 (12.7)	0.5	0 (0.0)	0.0
O42	79	4.3	56 (70.9)	3.1	20 (25.3)	1.1	3 (3.8)	0.2
O43	56	2.6	52 (92.9)	2.3	3 (5.4)	0.1	1 (1.8)	0.0
O46	13	0.6	11 (84.6)	0.5	2 (15.4)	0.1	0 (0.0)	0.0
O49	133	4.7	95 (71.4)	3.4	35 (26.3)	1.2	3 (2.3)	0.1
O52	80	3.3	56 (70.0)	2.3	24 (30.0)	1.0	0 (0.0)	0.0
O61	68	4.3	54 (79.4)	3.4	14 (20.6)	0.9	0 (0.0)	0.0
O63	67	3.7	54 (80.6)	3.0	13 (19.4)	0.7	0 (0.0)	0.0
O64	66	3.0	53 (80.3)	2.4	13 (19.7)	0.6	0 (0.0)	0.0
O68	98	4.1	81 (82.7)	3.4	16 (16.3)	0.7	1 (1.0)	0.0
O69	32	3.6	30 (93.8)	3.4	2 (6.3)	0.2	0 (0.0)	0.0
O72	58	2.6	50 (86.2)	2.2	8 (13.8)	0.4	0 (0.0)	0.0
O77	66	4.4	59 (89.4)	4.0	7 (10.6)	0.5	0 (0.0)	0.0
J45	53	3.6	45 (84.9)	3.0	7 (13.2)	0.5	1 (1.9)	0.1
J60	45	3.0	43 (95.6)	2.9	2 (4.4)	0.1	0 (0.0)	0.0
J62	31	3.1	24 (77.4)	2.4	7 (22.6)	0.7	0 (0.0)	0.0
J69	15	1.3	9 (60.0)	0.8	6 (40.0)	0.5	0 (0.0)	0.0
J72	30	2.9	27 (90.0)	2.6	3 (10.0)	0.3	0 (0.0)	0.0
J74	14	4.6	9 (64.3)	3.0	5 (35.7)	1.7	0 (0.0)	0.0
J99	27	2.9	27 (100.0)	2.9	0 (0.0)	0.0	0 (0.0)	0.0
Sum	1776	-	1478	-	279	-	19	-
Mean	48	3.0	42 (84.1)	2.5	8 (15.3)	0.5	1 (0.7)	0.0
s.d.	27	1.1	237 (9. 8)	0.9	45 (9.4)	0.4	3 (2.2)	0.1

a Percentage of total ingestions.

## Discussion

### PDER event detection and possible sources of variation

Through the use of multivariate statistical analysis coupled with laboratory simulations of ingestion events, we have demonstrated that stomach temperature telemetry allows a fine discrimination between the three possible ingestion types (*i.e.* milk, seawater, and solid food) in harbour seal pups. The present study also confirmed, in accordance with previous work [29], that the retention time of the STP, which is usually the limiting factor when using the stomach temperature telemetry technique in several adult species, is not a problem in pre-weaned harbour seals. Indeed, most pups from which equipment was recovered (95%) retained the device throughout the STP battery lifespan (∼21 d), while the STP either stopped transmitting or was lost during the recording period on only 2 occasions (Table 1).

Another challenge related to the use of stomach temperature telemetry to monitor animal foraging behaviour is to accurately detect the ingestions in the temperature records [42], [47], [56], [62]. The visual clarity of the temperature records and subsequent ease in identifying the PDER events varied between individuals. This might be attributable to numerous factors, including differential position of the STP in the stomach and/or the individual's activity level [46]. Also, since milk ingestions result in stomach temperature drops of considerably lower amplitude than fish or water ingestions, they are harder to discriminate from stomach temperature background variations. Nevertheless, in this study, most PDER events (72.1%) were visually identified with great confidence.

Of the 496 PDER events that could not be visually classified with high confidence, 113 (22.8%) did not exhibit the typical exponential recovery. This pattern has also been reported in wandering albatross stomach temperature records [46]. PDER events from albatrosses displaying unexpected recovery rates nonetheless resulted from prey ingestions [46], suggesting that those identified in the present study likely arose from true ingestions. As to the slowly decreasing PDER events (*n* = 29), all classified as milk ingestions, they might be attributable to particularly slow suckling, which would affect the rate of stomach filling and subsequent heating process, as suggested by [28].

Because of the numerous factors affecting the rate and amplitude of stomach temperature changes following ingestion in endotherms, validation studies are required prior to any interpretation of stomach temperature data [63]. Despite our inability to observe ingestion behaviour coinciding with recorded PDER events in free-ranging harbour seal pups, ingestion simulations carried out in the laboratory resulted in PDER events sufficiently similar to those extracted from records on living animals to visually and statistically classify them as either milk, seawater or solid food ingestions. Furthermore, the PDER events described in this study were very similar to those obtained from a captive harbour seal pup where suckling observations were performed [28], providing support to our interpretation of the results.

### PDER event classification

The cluster analysis produced numerous groups for both milk and colder material ingestions (3 groups for milk, and 2 mixed groups containing seawater and solid food ingestions), which is likely attributable to the important intra- and inter-individual variations in stomach temperature in absence of PDER events (as previously described in avian and pinniped species; *e.g.*, [46], [47]), amount of material ingested, suckling rate and whether nursing took place in the water or on land. Nonetheless, once groups 1, 3 and 5 and groups 2 and 4 were pooled together, the clustering procedure allowed the successful discrimination between milk and colder material ingestions (*i.e.*, seawater and solid food). No further discrimination could be achieved by this technique, and such limited resolution is likely due to the small proportion (1.1%) of PDER events representing solid food ingestions.

The ingestion simulations carried out in the laboratory provided the information needed to efficiently discriminate between PDER events deemed seawater and solid food ingestions. PDER events resulting from the injection of material into the simulated stomachs exhibited greater temperature drop amplitudes and lower recovery rates than equivalent PDER events recorded on living animals because the pup's stomach mixing and heat conductivity were not measured and imitated [28]. However, the general patterns of water and fish injections were in agreement to those expected (Fig. 1B), allowing comparisons between the curve characteristics of the PDER events simulated in the laboratory and those recorded on living animals. The negative discriminant coefficient generated from the LDA for the quantity of material injected in the simulated stomach confirms that this variable has much less of an effect on PDER event characteristics than the thermal properties of water and fish. The retention of the *I* index as the best predictor of ingestion type for PDER events resulting from water or fish injection in the simulated stomach is consistent with the previous use of this variable to accurately discriminate feeding from seawater ingestions in adult seabirds and pinnipeds [41], [47].

By using the *I* index distribution, we were also able to correctly re-assign some PDER events (5.6%) that were misclassified as milk ingestions by the cluster analysis method into the seawater ingestion category. Such secondary classification appears reliable since most of the re-assigned PDER events were deemed seawater ingestions in the preliminary visual classification and were classified by the K-means in the left and higher portion of group 1 (Fig. 3), which appears to be continuous with the distribution of the groups mostly containing seawater ingestions (groups 2 and 4). Despite this, some PDER events (*n* = 43) from group 1 with particularly great *T_A_-T_B_* values remained classified as milk ingestions, which explains the residual discrepancy (2.4%) between the preliminary visual and statistical classification approaches. These PDER events might have been generated by underwater nursing events where pups would have ingested small amounts of seawater while suckling or by ingestion of small volumes of solid prey, but were nonetheless considered as milk ingestions.

Finally, the addition of the multiple and low confidence PDER events to the classified ones by means of the LDA allowed their classification without biasing the initial discrimination process. The categorization of the multiple and low confidence events could only be compared to the preliminary visual classification, which revealed minimal discrepancy between the two approaches. Taken together, the multivariate statistical analysis, simulated ingestions, preliminary visual classification and *a posteriori* LDA allowed the classification of all PDER events recorded in free ranging harbour seal pups with high reliability. It therefore appears that stomach temperature telemetry represents an interesting method to monitor nursing and feeding behaviour in wild pinniped pups.

The detection of ingestions using stomach temperature monitoring requires that ingested items and animal's body temperatures differ. Likewise, the identification of items ingested among multiple possibilities involves that items differ in their physical properties (*i.e.* volume ingested, temperature and thermodynamic properties). Therefore, temperature telemetry represents a promising method to study consumption behaviour in early life and transition from parental provisioning to nutritional independence in all endotherm species where ingested items by offspring before and after fledging/weaning fulfill these requirements.

### Seawater ingestion and its possible causes

Seawater ingestion in pre-weaned harbour seal pups could result from side-effects of aquatic nursing [29] or prey capture attempts. Alternatively, its high occurrence (0–40% of the PDER events, *n* = 35 pups) might suggest that mariposia (seawater drinking) represents a common behaviour in pre-weaned harbour seal pups and that it might serve a physiological or ecological role.

Mariposia was shown to naturally occur in many pinniped species of all age classes [64], [65], [66], [67], [68]. Although the causes of mariposia have not been extensively investigated in free-ranging pinniped pups, such behaviour might serve physiological functions, such as restoration of water balance when experiencing dehydration [69] or when facing long periods without maternal milk supply. Lydersen *et al*. [70] suggested that snow ingestion by ringed seal pups during lactation might be related to dehydration, and Schreer *et al*. [29] found that seawater drinking mostly occurred in harbour seal pups that lost weight. Since the kidneys of some phocid species might have the capacity to excrete the extra salt from seawater to provide a net water gain from seawater drinking [66], the hypothesis of harbour seal pups drinking seawater to restore water balance when dehydrated was considered. However, we did not observe that the time since the last nursing event was greater for seawater ingestion than for milk ingestion, which suggests that it generally occurs within the usual interval between two suckling events. Moreover, the individual mean daily frequency of seawater ingestion was not correlated with pup growth rate during the lactation period, which indicates that the pups ingesting seawater were not limited in their energy input and that dehydration may not be the explanation for this behaviour.

These results, together with the findings that pups were in the water during most ingestions occurring after seawater ingestions, suggest that seawater ingestions might arise as incidental side-effects of suckling attempts in the water. This is further supported by the highly reduced time before the milk ingestion following seawater ingestions compared to that for milk ingestions. Similarly, a captive harbour seal pup equipped with a TDR and a STP was observed attempting but failing to suckle in the water [28]. Following these attempts, the mother-pup pair sometimes hauled-out and nursed within few minutes. However, as free ranging harbour seal pups are mainly nursed in the water [29], they may incidentally swallow small amounts of seawater while suckling. The increasing relative occurrence of seawater ingestion throughout the first 3 weeks after birth (Fig. 5) could therefore be attributable to underwater suckling attempts increasingly being rejected by the female as part of the weaning process.

### Solid food ingestion

In most phocid species, weaning is thought to be abrupt and followed by a post-weaning fast during which pups develop their capacities to forage on their own [12], [13], [14], [71]. However, the detection of solid food ingestions from 12 *d* of age implies that some harbour seal pups begin consuming prey while still being nursed, as reported in most otariid species [12]. This result is also supported by our observations of several pre-weaned pups producing unusually green-colored feces sometimes containing traces of solid items which are unlikely to result from a diet solely based on maternal milk.

Since solid food ingestions were recorded in 20% (*n* = 7) of the studied pups, pre-weaning prey consumption might represent a tactic of energy allocation during the lactation period. As post-weaning survival depends on the rapid development of foraging abilities before complete depletion of the pre-weaning energy reserves [12], harbour seal pups might face a trade-off between passive energy storage and active development of prey capture abilities during lactation, as suggested in some otariid species (*e.g*., [72], [73]). The alternative tactic hypothesis is supported by our finding that pups which ingested solid food before weaning nursed more in the water than other pups, suggesting greater aquatic activity for the former.

Solid food ingestions represented only 1.1% of the PDER events recorded in this study. However, this proportion might not be representative of capture attempts since missing prey may result in seawater ingestions instead of solid food ingestions. Likewise, the ingestion of very small prey items accompanied by seawater could also result in a temperature drop typical of seawater. Thus, the increasing occurrence of seawater ingestion throughout the first 3 weeks of lactation may also be attributable to an increase in pre-weaning foraging activity.

Harbour seal pups sometimes accompany their mother during feeding trips at sea [23] and gradually improve their diving capacities in the weeks following birth so they can perform dives associated with feeding in adults before weaning [24], [74], [75]. It is thus conceivable that a proportion of them learn to forage prior to weaning, which is in accordance with the observations of pre-weaned Weddell seal pups bringing fish to the surface [76].

It was not possible to identify prey items ingested by harbour seal pups during the lactation period. However, it is likely that harbour seal's initial diet is composed of small pelagic crustaceans, a major early source of solid food consumed by all phocid species studied so far [12]. Weddell seal pups dive from 2 weeks of age [77] and there is evidence that they start feeding on crustaceans before weaning [78]. A mixture of milk and crustaceans has also been reported in stomachs from bearded and ringed seal pups approaching weaning, two other phocid species exhibiting precocious swimming activities [79]. Similarly, crustaceans were found abundant in newly weaned harbour seal pups' stomachs [49] and small fish (average 4–8 cm long) such as sand lance, silver hake and American plaice appear to play an important role in harbour seal pups' early diet [71].

In summary, the present study has demonstrated that stomach temperature telemetry provides an effective means of qualifying the consumption behaviour of harbour seal pups throughout their lactation period. The application of multivariate statistical approaches using stomach temperature variables and comparison with simulated ingestions allowed a sensitive classification of the 3 possible ingestion types: milk, seawater and solid food. This led to new insights into the consumption ontogeny of this precocious phocid species. The ingestion of solid food prior to weaning raises questions about a possible trade-off in pups' energetic budget during the lactation period and suggests that weaning can be less abrupt in harbour seals than among most phocids. This study provides valuable information which may help unravel the determinants of pre-weaning growth and post-weaning survival in precocious phocid species, and also presents a promising method to study the transition to nutritional independence in various endotherms.
